# GPR3 Stimulates Aβ Production via Interactions with APP and β-Arrestin2

**DOI:** 10.1371/journal.pone.0074680

**Published:** 2013-09-12

**Authors:** Christopher D. Nelson, Morgan Sheng

**Affiliations:** Department of Neuroscience, Genentech, Inc., South San Francisco, California, United States of America; Massachusetts General Hospital, United States of America

## Abstract

The orphan G protein-coupled receptor (GPCR) GPR3 enhances the processing of Amyloid Precursor Protein (APP) to the neurotoxic beta-amyloid (Aβ) peptide via incompletely understood mechanisms. Through overexpression and shRNA knockdown experiments in HEK293 cells, we show that β-arrestin2 (βarr2), a GPCR-interacting scaffold protein reported to bind γ-secretase, is an essential factor for GPR3-stimulated Aβ production. For a panel of GPR3 receptor mutants, the degree of stimulation of Aβ production correlates with receptor-β-arrestin binding and receptor trafficking to endocytic vesicles. However, GPR3’s recruitment of βarr2 cannot be the sole explanation, because interaction with βarr2 is common to most GPCRs, whereas GPR3 is relatively unique among GPCRs in enhancing Aβ production. In addition to β-arrestin, APP is present in a complex with GPR3 and stimulation of Aβ production by GPR3 mutants correlates with their level of APP binding. Importantly, among a broader selection of GPCRs, only GPR3 and prostaglandin E receptor 2 subtype EP2 (PTGER2; another GPCR that increases Aβ production) interact with APP, and PTGER2 does so in an agonist-stimulated manner. These data indicate that a subset of GPCRs, including GPR3 and PTGER2, can associate with APP when internalized via βarr2, and thereby promote the cleavage of APP to generate Aβ.

## Introduction

Alzheimer’s Disease (AD) is a progressive neurodegenerative disorder estimated to affect ∼5 million people in the United States and approximately 36 million people worldwide, with numbers predicted to grow further as a result of an aging global population [1]–[3]. Recent advances in molecular pathology and human genetics have reinforced the amyloid hypothesis for the etiology of AD: that the accumulation of Aβ peptide (produced by cleavage of APP by BACE1 and the γ-secretase complex) is the key initiator of AD pathogenesis [4]–[7]. For wild-type APP, cleavage by BACE1 is the rate-limiting step in Aβ production [8], but with some mutations found in familial AD – for example, the K670N/M671L APP695 (Swedish APP) mutant – APP is more readily processed by BACE and the production of Aβ is enhanced [9], [10]. In addition to efforts to develop clinically useful drugs that inhibit BACE and γ-secretase [11]–[13], researchers’ attention has also been drawn to indirect modulators of APP processing with a goal of uncovering new potential therapeutic targets.

A cDNA screen for modulators of APP processing uncovered the effects of GPR3 [14], an orphan GPCR most highly expressed in the brain, ovaries and testes [15], [16]. GPR3 is a constitutively active G_s_-coupled receptor that activates adenylyl cyclase, raising intracellular cAMP [17], [18]. Thathiah et al showed GPR3 potentiates γ-secretase activity and stimulates the production of Aβ1-40 and 1-42 in transfected neurons [14]. Further, the authors found a gene dosage-dependent effect of GPR3 on Aβ production in vivo, as wild-type, GPR3 heterozygous knockouts and GPR3 homozygous knockouts showed a progressive reduction in soluble Aβ levels in hippocampus [14]. Interestingly, although GPR3 exhibits a high constitutive G protein coupling, effects of the receptor on Aβ production were independent of G_s_ and cAMP signaling [14].

The finding that GPR3-stimulated APP processing is a G protein-independent process led us to hypothesize that this signaling pathway may involve the β-arrestins. The two β-arrestin isoforms, β-arrestin1 (βarr1) and β-arrestin2 (βarr2), are ubiquitously expressed adaptor proteins that are recruited to activated GPCRs [19]. Originally the β-arrestins were discovered as key modulators of homologous receptor desensitization, a process that antagonizes the G protein coupling of agonist-occupied GPCRs via phosphorylation by the G protein-coupled receptor kinases (GRKs), leading to β-arrestin recruitment and steric hindrance of G protein activation [20], [21]. However, β-arrestins are also essential for endocytosis of receptors via clathrin-coated pits through interactions with clathrin [22] and AP-2 adaptor protein [23]. More recently, it has been shown that β-arrestins coordinate several G protein-independent GPCR signaling cascades [24]–[26]. In these cases, the β-arrestin typically serves as a molecular scaffold, assembling multiple elements of a signaling cascade at activated receptors, thereby regulating the temporal and spatial activity of the pathway.

Here we report that GPR3 can be found in a protein complex with APP, and this interaction is promoted by βarr2. Using a set of GPR3 mutants, we show that association of GPR3 with APP correlates with enhanced Aβ production, β-arrestin recruitment and localization of the receptor in endocytic vesicles. Testing a wider panel of GPCRs, we found all receptors interact with β-arrestins, but only GPR3 and PTGER2 showed appreciable interaction with APP and stimulated Aβ production. Thus, we propose that a subset of GPCRs is capable of forming a receptor-APP complex in a βarr2-dependent manner to facilitate the generation of Aβ.

## Experimental Procedures

### Materials and Reagents

The following reagents were purchased (vendor): Tissue culture media, Lipofectamine 2000 transfection reagent and Alexa dye-conjugated secondary antibodies (Life Technologies); anti-EEA1 (Bethyl Laborotories, Inc.); anti-APP (22c11) (Millipore); rabbit anti-β-arrestin1/2 (Cell Signaling, Inc); Protein A/G Plus resin and DSP crosslinking reagent (ThermoFisher); PhosSTOP phosphatase inhibitor tabs, protease inhibitor tablets, and β-octylglucoside (Roche); Aβ1-40 ELISA plates (MesoScale Discovery); HTRF kit for cAMP measurement (CisBio). All other reagents were purchased from Sigma.

### DNA Constructs and Cell Lines

To create the APP-HEK stable cell lines, HEK293 cells were transiently transfected with wild-type (WtAPP-HEK) or Swedish APP (SweAPP-HEK) in the pRK5 Neo vector using Lipofectamine 2000 according to manufacturer’s instructions and grown in the presence of 1 mg/mL G418 sulfate for clonal selection. After screening clonal populations for APP expression, selected cell lines were maintained with G418 at 400 μg/mL. Human GPR3, PTGER2 and M1AChR cDNAs were obtained from the Genentech cDNA core facility and subcloned into pcDNA3.1 zeo- (Life Technologies) with an N-terminal leader sequence [27] and FLAG-tag. FLAG-β1AR and FLAG-β2AR constructs were from Robert J. Lefkowitz at Duke University. Rat β-arrestin1 and β-arrestin2 cDNA were also from the Genentech cDNA core facility, subcloned into pcDNA3.1 zeo- and fused in-frame with a C-terminal EGFP tag. Constructs for shRNA knockdown of β-arrestin2 [28] and a firefly luciferase control [29] were created in the pSuper vector, using target sequences as previously described. All GPR3 mutants were created with a QuikChange Multi Site-Directed Mutagenesis Kit (Stratagene) and verified by DNA sequencing.

### Cell Culture and Aβ ELISA

WtAPP- and SweAPP-HEK cells were maintained in DMEM with 10% fetal calf serum and 1% penicillin/streptomycin and transfected using Lipofectamine 2000 according to manufacturer’s instructions. After 72 hours, the culture media was collected and centrifuged at 14,000× g for 10 minutes at 4°C to remove cell debris. The amount of Aβ1-40 in the supernatant was quantified using an MSD ELISA kit, corrected for protein content of the corresponding lysate to adjust for differences in cell density, and normalized to vector controls for each stable cell line. For GPCR agonist experiments, the transfected cells were serum-starved in DMEM +0.4% BSA for at least 1 hour before stimulation.

### Co-Immunoprecipitation Assays

SweAPP-HEK cell lines were maintained and transfected as described above. At 48–72 hours post-transfection, the cells were washed twice with ice-cold PBS, lysed in RIPA buffer supplemented with 1% β-octylglucoside, protease and phosphatase inhibitors and tumbled at 4°C. Samples were normalized for total protein concentration and 1 mg per sample was used for immunoprecipitation (IP) with M2 anti-FLAG beads or 22c11 anti-APP followed by protein A/G beads. The beads were washed four times with cold RIPA buffer and incubated with Laemmli sample buffer before separation by SDS-PAGE. For endogenous β-arrestin co-immunoprecipitation with GPCRs, cells were treated with DSP crosslinking reagent before lysis, as previously described [30].

### Immunocytochemistry and Image Acquisition

SweAPP-HEK cells or dissociated rat hippocampal cultures (DIV21) were grown on glass coverslips, transfected with the indicated plasmids using Lipofectamine 2000 and fixed in PBS with 4% paraformaldehyde and 4% sucrose 72 hours later. Cells were incubated with primary antibodies in GDB buffer (30 mM phosphate buffer, pH 7.4, containing 0.1% gelatin, 0.3% Triton X-100, and 0.45 M NaCl) at 4°C overnight, washed three times in PBS at room temperature, and labeled with Alexa-conjugated secondary antibodies, followed by another three washes and mounting on glass microscope slides. Images were acquired on a Leica SP5 confocal microscope and binned according to GPR3 subcellular localization with the operator blinded to the transfection conditions. For analysis of receptor clustering, the blinded observer binned transfected cells into clustered or dispersed phenotypes and a minimum of 30 transfected cells were counted per transfection to determine the representative percentages for a given sample, prior to unblinding.

### Statistical Analysis

Statistical significance was determined with the aid of Graphpad Prism software using one-way ANOVA and either a Bonferroni (multiple comparisons) or Dunnett (comparing all sets with control) post-hoc test as indicated. All values shown are the mean +/− SEM.

## Results

**Figure 1:**
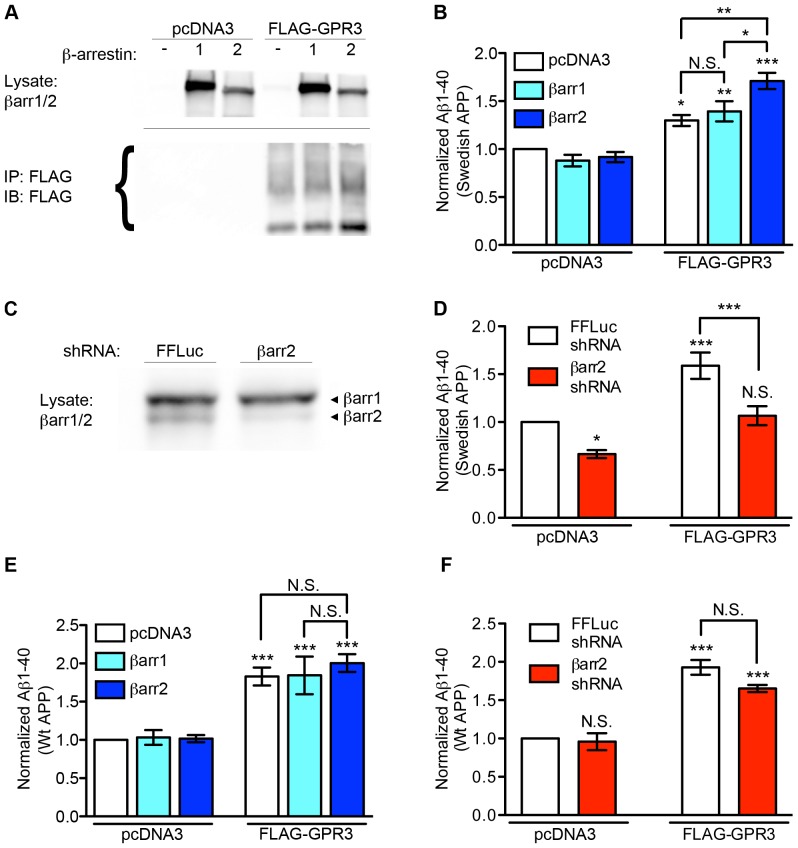
βarr2 influences GPR3-mediated Aβ production in SweAPP-HEK cells. A). Representative Western blots of overexpressed βarr1 and βarr2 (upper) and GPR3 (lower) in cell lysates and FLAG IPs from SweAPP-HEK cells transfected as indicated. Brackets indicate the multiple bands corresponding to GPR3. B). Aβ1-40 was measured by ELISA in culture supernatant from SweAPP-HEK cells transfected with empty vector, βarr1-EGFP or βarr2-EGFP in the presence or absence of co-transfected FLAG-GPR3. n = 8, 7, 11, 8, 6 and 7 independent transfections from left to right. C). Representative Western blot of endogenous β-arrestins from SweAPP-HEK cells transfected with control (FFLuc) or βarr2 shRNA. D) Aβ1-40 levels were measured from culture supernatants of SweAPP-HEK293 cells transfected with shRNA plasmids and either empty vector or FLAG-GPR3 as indicated. n = 8, 10, 7, and 10 replicates from left to right. E). Aβ ELISA results from WtAPP-HEK cells transfected with GPR3 and βarr1 or βarr2, as indicated. n = 6, 4, 6, 6, 4 and 6 from left to right. F). Aβ levels from WtAPP-HEK cells transfected with luciferase or βarr2 shRNA plus FLAG-GPR3 or vector, as indicated. n = 4 for all transfection conditions. Statistical analyses were performed by one-way ANOVA with a Bonferroni post-hoc test comparing all groups with vector-only/luciferase control, and selected comparisons as indicated (*p<0.05, **p<0.01, ***p<0.001).

Transient transfection of FLAG-tagged GPR3 into HEK cells stably transfected with the Swedish mutant of APP (SweAPP-HEK cells) (Figure 1A) boosted Aβ1-40 production by ∼30% (1.30+/−0.06 fold; as measured by ELISA) relative to vector control (Figure 1B). In the absence of GPR3 co-expression, transfection of neither β-arrestin1 (0.88+/−0.06) nor β-arrestin2 (0.91+/−0.05) had a significant effect on Aβ1-40 levels in the culture media. Co-transfection of βarr1 with GPR3 did not significantly increase Aβ production beyond GPR3 alone (1.39+/−0.10). In contrast, co-transfection of βarr2 further potentiated the Aβ production induced by GPR3 (1.71+/−0.08) (Figure 1B). To corroborate this observation, we used shRNA selective for β-arrestin2 to knock down endogenous βarr2 in our SweAPP-HEK cells. Consistent with previously published results [28], transfection of βarr2 shRNA depleted β-arrestin2 by ∼80% relative to a control shRNA targeting firefly luciferase, without affecting βarr1 levels (Figure 1C). Knockdown of βarr2 lowered basal Aβ (0.67+/−0.04) and also suppressed GPR3-induced Aβ production (1.07+/−0.10) (Figure 1D). Collectively, these data confirm that GPR3 is capable of enhancing Aβ production and show that β-arrestin2 is important for this effect in SweAPP-HEK cells.

We also conducted β-arrestin overexpression experiments in a HEK cell line stably expressing wild-type APP (WtAPP-HEK) (Figure 1E). While the absolute levels of Aβ produced were lower than those from SweAPP-HEK cells, GPR3 also enhanced Aβ production in WtAPP-HEK cells (1.83+/−0.12 fold, compared with WtAPP-HEK transfected with empty vector). However, overexpression of βarr2 with GPR3 failed to further enhance Aβ levels in these cells (2.00+/−0.11). Knockdown of βarr2 in WtAPP-HEK cells showed a trend toward reducing Aβ production when co-transfected with GPR3 (1.65+/−0.05 versus 1.93+/−0.10 fold for GPR3 plus control shRNA), but this did not reach statistical significance (Figure 1F). Thus we conclude that GPR3 is capable of boosting wild-type APP processing, but the GPR3-enhancing effects of βarr2 are only uncovered with the Swedish APP mutant.

**Figure 2:**
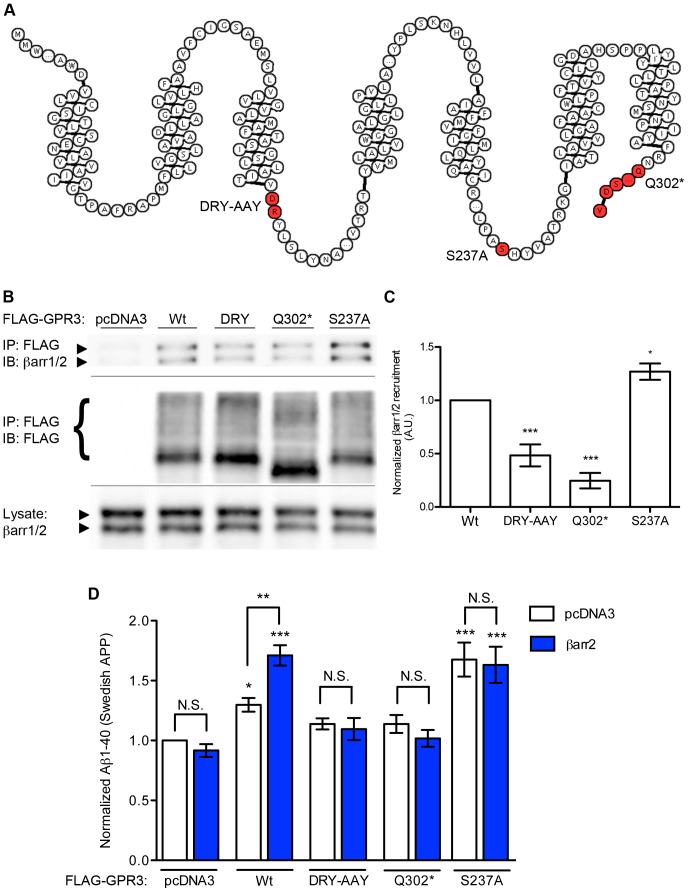
GPR3 mutants alter Aβ production and β-arrestin binding. A). Schematic diagram of GPR3 highlighting in red the mutations used in this study. The diagram was made using the web-based RdBe service [43]. B). Co-immunoprecipiation of endogenous β-arrestins from SweAPP-HEK cells with transfected FLAG-tagged GPR3 (wild-type and mutants). C). Quantification of β-arrestin co-IP with FLAG-GPR3 mutants. n = 5, 5, 5 and 4 independent experiments from left to right. Statistical significance was determined by one-way ANOVA with a Dunnett test comparing all mutants with wild-type GPR3 (*p<0.05, ***p<0.001). D). ELISA measurements of Aβ1-40 from SweAPP-HEK293 cells transfected with GPR3 mutants and βarr2 as indicated. Data sets for empty vector and wild-type GPR3 are re-plotted from Figure 1B. n = 8, 11, 8, 7, 10, 7, 4, 3, 4 and 3 from left to right. Statistical analyses were performed by one-way ANOVA with a Bonferroni post-hoc test comparing all columns with vector-only control, and selected comparisons as indicated (*p<0.05, **p<0.01, ***p<0.001).

Next, we used GPR3 mutants to alter the receptor-β-arrestin interaction and assess the importance of β-arrestin binding on GPR3-stimulated Aβ production (Figure 2). We investigated several mutations in the intracellular domains of GPR3: (i) DRY-AAY, a double point mutant at the base of the third transmembrane domain that impairs the G_s_-coupling of many GPCRs [31]–[34] and reduces GPR3-stimulated cAMP by >85% (Figure S1); (ii) Q302*, a mutant truncated after the seventh transmembrane segment, eliminating the intracellular carboxy-terminal tail required for efficient β-arrestin recruitment to most GPCRs [35]; and (iii) S237A, a point mutant in the third intracellular loop which removes a putative GRK site (Figure 2A). We examined GPR3-β-arrestin interaction by immunoprecipating the wild-type or mutant receptor (using FLAG antibody) and immunoblotting for co-immunoprecipitated endogenous β-arrestin proteins (Figure 2B, C). Normalized to wild-type GPR3, both the DRY-AAY (0.48+/−0.10) and Q302* (0.25+/−0.07) mutants showed reduced recruitment of β-arrestins. Unexpectedly, the S237A mutation resulted in significantly increased β-arrestin association with GPR3 (1.27+/−0.08 fold).

We also measured the effect of each GPR3 mutant on Aβ production, with and without co-transfected βarr2 (Figure 2D). DRY-AAY in our cell line did not significantly alter Aβ production (1.14+/−0.05), and overexpression of βarr2 with this mutant made no difference (1.10+/−0.9). Deletion of the carboxyl tail in the Q302* mutant also prevented the GPR3-mediated Aβ increase (1.14+/−.07 fold relative to control for Q302* plus empty vector, and 1.02+/−.7 fold when Q302* was co-transfected with βarr2). The S237A mutant stimulated Aβ production to a stronger degree than wild-type GPR3, either without (1.68+/−.14) or with βarr2-EGFP (1.63+/−.15). Thus, the strength of association with β-arrestins correlates with the Aβ production of the GPR3 variants (S273A>Wt>DRY-AAY = Q302*).

**Figure 3:**
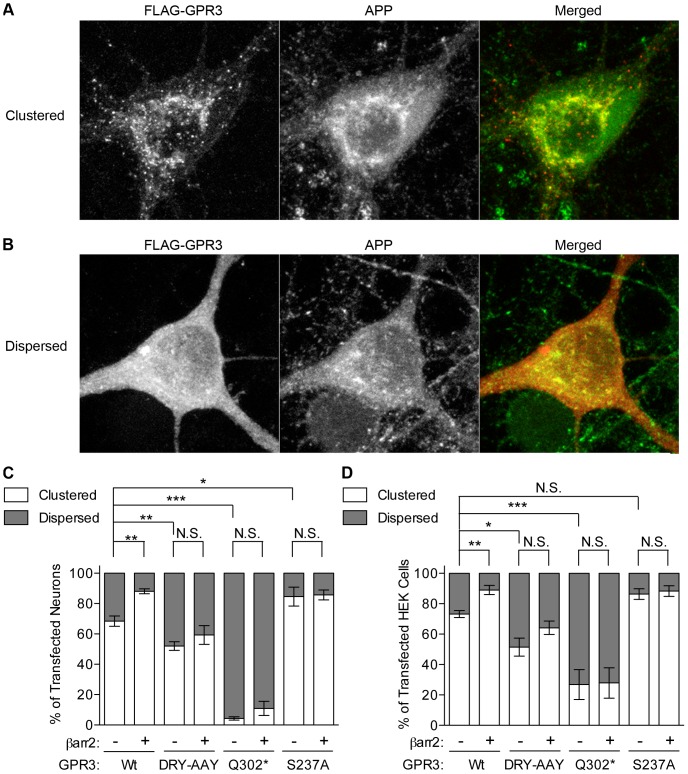
GPR3 mutants show differences in subcellular localization. A). Representative confocal images of the “clustered” GPR3 distribution pattern in transfected rat hippocampal neuron culture (DIV20+3). Left to right are immunostaining for FLAG (GPR3 wild-type and mutants), endogenous APP and a merged pseudocolor composite of the two channels (FLAG in red, APP in green). B). Representative confocal images of the “dispersed” GPR3 pattern in transfected rat hippocampal neuron culture (DIV20+3). C). Quantification of clustered and dispersed phenotypes in transfected neurons for the GPR3 mutants in the presence or absence of co-transfected βarr2-EGFP. n = 3 independent transfections for each condition. D). Quantification of clustered and dispersed phenotypes in transfected SweAPP-HEK cells for the GPR3 mutants in the presence or absence of co-transfected βarr2-EGFP. n = 5 independent transfections for each condition. Statistical significance was determined by one-way ANOVA with a Bonferroni post-hoc test comparing clustering in all conditions with GPR3 alone, and selected comparisons as indicated (*p<0.05, **p<0.01, ***p<0.001).

We next examined the subcellular localization of GPR3 in neurons. Mature rat hippocampal neurons were transfected with FLAG-tagged GPR3 after 20 days in culture and immunostained with anti-FLAG antibody 3 days later (DIV20+3). In the majority of cells (68+/−3%), wild-type FLAG-GPR3 was observed as intracellular clusters that showed a high degree of co-localization with endogenous APP (Figure 3A). We found that the GPR3 clusters also partially co-localized with endogenous β-arrestins in many neuronal cell bodies (Figure S2A) as well as with the early endosomal marker EEA1 (Figure S2B), the latter being consistent with fractionation experiments in previous studies [36]. In the remainder of the transfected neurons, FLAG-GPR3 showed a more diffuse staining pattern (Figure 3B). Co-transfection of βarr2-EGFP enhanced the “clustered” pattern of co-transfected FLAG-GPR3, such that 88+/−2% of transfected neurons showed this distribution (Figure 3C). These data suggest that overexpressed GPR3 is largely present in endosomal compartments and co-overexpression of βarr2 promotes GPR3 localization in these compartments, where the receptor can be found in proximity to APP.

GPR3 mutants transfected into rat hippocampal neurons also exhibited the same “clustered” or “dispersed” distribution patterns as wild-type GPR3, though in different proportions (Figure 3C). DRY-AAY-GPR3 was slightly but significantly less likely to be clustered in neurons than wild-type GPR3 (52+/−3% of transfected neurons showed a clustered GPR3 pattern). The Q302* mutation greatly impaired clustering with only 4+/−1% of neurons showing a punctate GPR3-Q302* distribution pattern. On the other hand, the S237A mutant showed enhanced clustering relative to wild-type, with 85+/−6% of transfected cells exhibiting this pattern. Unlike wild-type GPR3, co-transfection of additional β-arrestin2 did not significantly affect the receptor distribution phenotypes for the GPR3 mutants (Figure 3C). We also found similar results with the subcellular distribution patterns of GPR3 (wild-type and mutants) transfected in SweAPP-HEK293 cells (Figure 3D). Here, as in transfected neurons, we observed a rank order of intracellular clustering (S237A>wild-type GPR3> DRY-AAY>Q302*). Also similar to neurons, only wild-type GPR3 was enhanced in clustering by co-transfection of βarr2 (Wt+pcDNA3, 73+/−2% vs. Wt+βarr2, 89+/−3%). DRY-AAY (51+/−6% vs. 64+/−4%) showed a trend towards increase that did not reach significance, while Q302* (29+/−9% vs. 29+/−10%) and S237A (86+/−4% vs. 88+/−4%) were indistinguishable between empty vector and βarr2-EGFP co-transfections. These data indicate that, as with β-arrestin recruitment, accumulation of GPR3 in endosomal compartments follows the rank order S237A>Wt>DRY-AAY>Q302*, which correlates with the rank order for stimulation of Aβ production.

**Figure 4:**
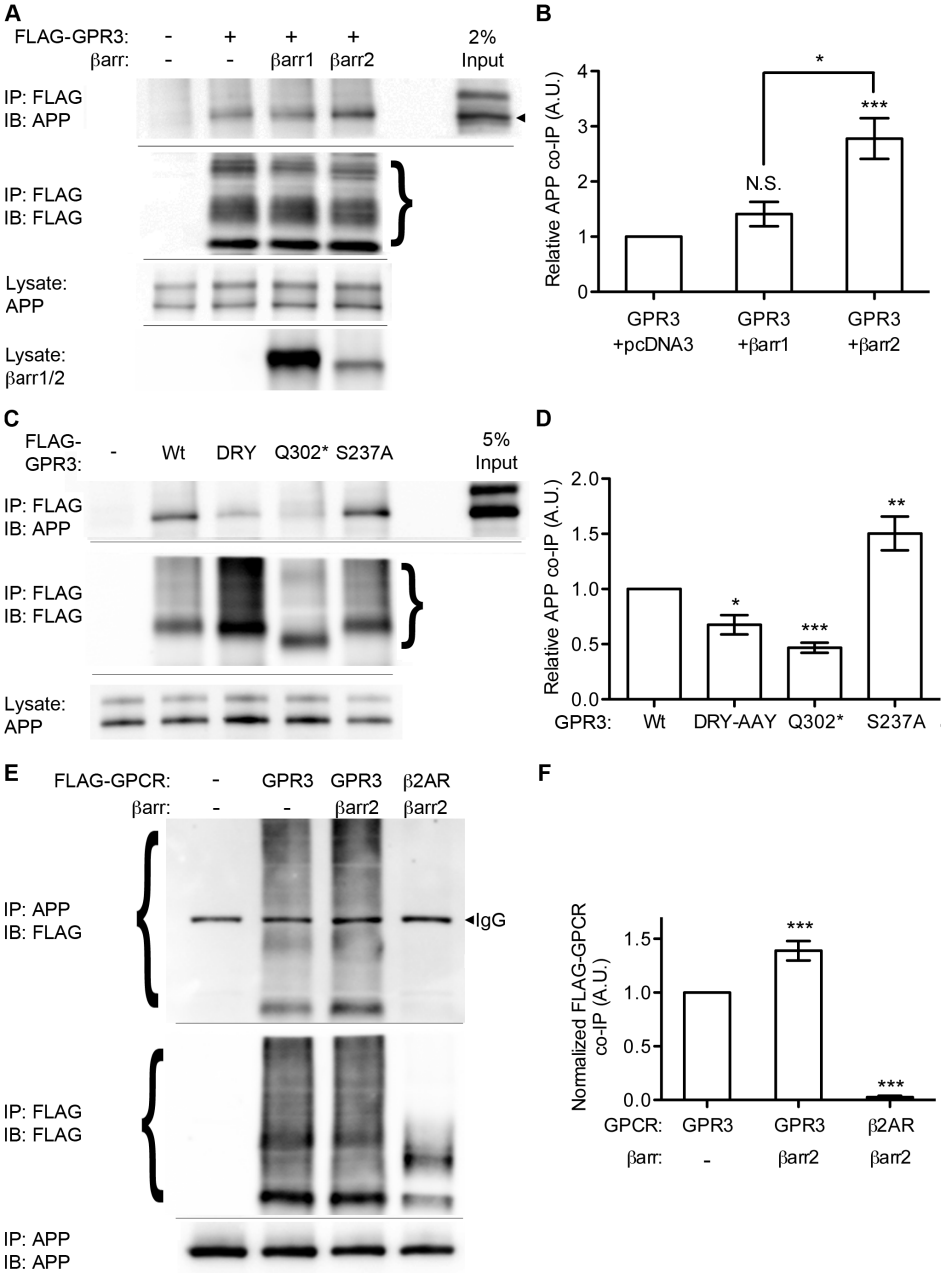
Biochemical association of GPR3 and APP is stimulated by β-arrestin. A). SweAPP-HEK cells, transfected as indicated, were immunoprecipitated with FLAG antibody and immunoblotted for APP or FLAG. Arrowhead indicates the band for APP. B). Quantification (by densitometry) of co-IP of APP with FLAG-GPR3. n = 6, 3 and 5 from left to right. Statistical significance was determined by one-way ANOVA with a Bonferroni post-hoc test comparing all data sets (*p<0.05, ***p<0.001). C). Co-immunoprecipitation of APP with FLAG-GPR3 mutants. SweAPP-HEK cells were transfected as indicated and blotted for immunoprecipitated FLAG and co-immunoprecipitated APP as indicated. D) Densitometry for APP co-immunoprecipitated with FLAG-GPR3 constructs. n = 6, 6, 6 and 5 independent experiments left to right. Statistical significance was determined by one-way ANOVA with a Dunnett test comparing all columns with wild-type GPR3. (*p<0.05, **p<0.01, ***p<0.001). E). GPR3, but not β2AR, co-immunoprecipitates with APP. SweAPP-HEK cells were transfected as indicated and lysates immunoprecipitated as indicated. Arrowhead indicates the band for heavy-chain IgG from the 22c11 (anti-APP) IP reaction. F). Quantification of FLAG-GPCR co-IP with APP. n = 5 for all data sets. Statistical significance was determined by one-way ANOVA with a Dunnett test comparing all columns with wild-type GPR3. (***p<0.001).

Having observed GPR3 co-localization with APP, we asked whether these two molecules could be found together in a protein complex. Because overexpression of βarr2 was found to enhance both Aβ production and receptor clustering, we also tested whether β-arrestins influenced the formation of such a GPR3-APP complex. We transfected SweAPP-HEK cells with FLAG-tagged GPR3 plus empty vector control, βarr1-EGFP, or βarr2-EGFP and examined FLAG IPs for co-immunoprecipitation of APP (Figure 4A–B). With FLAG-GPR3, but not vector control, appreciable amounts of full-length APP were co-immunoprecipitated with FLAG antibodies. Relative to GPR3 alone, co-transfection of βarr1 with GPR3 did not significantly increase the amount of APP co-immunoprecipitated with the receptor. On the other hand, co-transfection of βarr2 enhanced GPR3-APP association, resulting in 2.78+/−0.37 fold more APP co-precipitated than with GPR3 alone. We are unable to tell from these experiments whether the GPR3-APP interaction is direct or indirect. However, it does not appear to be mediated solely through β-arrestin2 binding, as APP was not found to co-immunoprecipitate with β-arrestins pulled down via their EGFP tag (Figure S3).

Do the GPR3 mutations that affect β-arrestin binding and stimulation of Aβ production also show differences in association with APP? To answer this question, FLAG-tagged wild-type GPR3, DRY-AAY, Q302*, and S237A mutants were expressed in SweAPP-HEK cells and immunoprecipitated 3 days post-transfection (Figure 4C, D). DRY-AAY showed reduced APP co-IP (0.68+/−0.09 relative to wild-type GPR3), while the interaction of Q302* with APP was even more robustly attenuated (0.47+/−0.05). In contrast, S237A showed an elevated interaction with APP (1.50+/−0.15 fold increase). Thus among the GPR3 variants, formation of a GPR3-APP complex is positively correlated both with β-arrestin recruitment and Aβ production.

We confirmed the biochemical association of GPR3 and APP by performing the reciprocal IP reaction, immunoprecipitating APP from SweAPP-HEK cells transfected with FLAG-GPR3 alone, FLAG-GPR3 plus βarr2-EGFP, or βarr2-EGFP with FLAG-β2-adrenergic receptor (β2AR; another G_s_-coupled GPCR). GPR3 was readily found in the APP immunoprecipitates and this interaction was enhanced by co-transfection of βarr2-EGFP (1.39+/−0.09 fold increase versus to GPR3 alone) (Figure 4E, F). Notably, β2AR was not detected above background in the APP immunoprecipitates (0.03+/−0.01 (Figure 4E, F). These data confirm that GPR3 can interact with APP, and does so with some specificity, insofar as another GPCR did not associate with APP under the same conditions.

**Figure 5:**
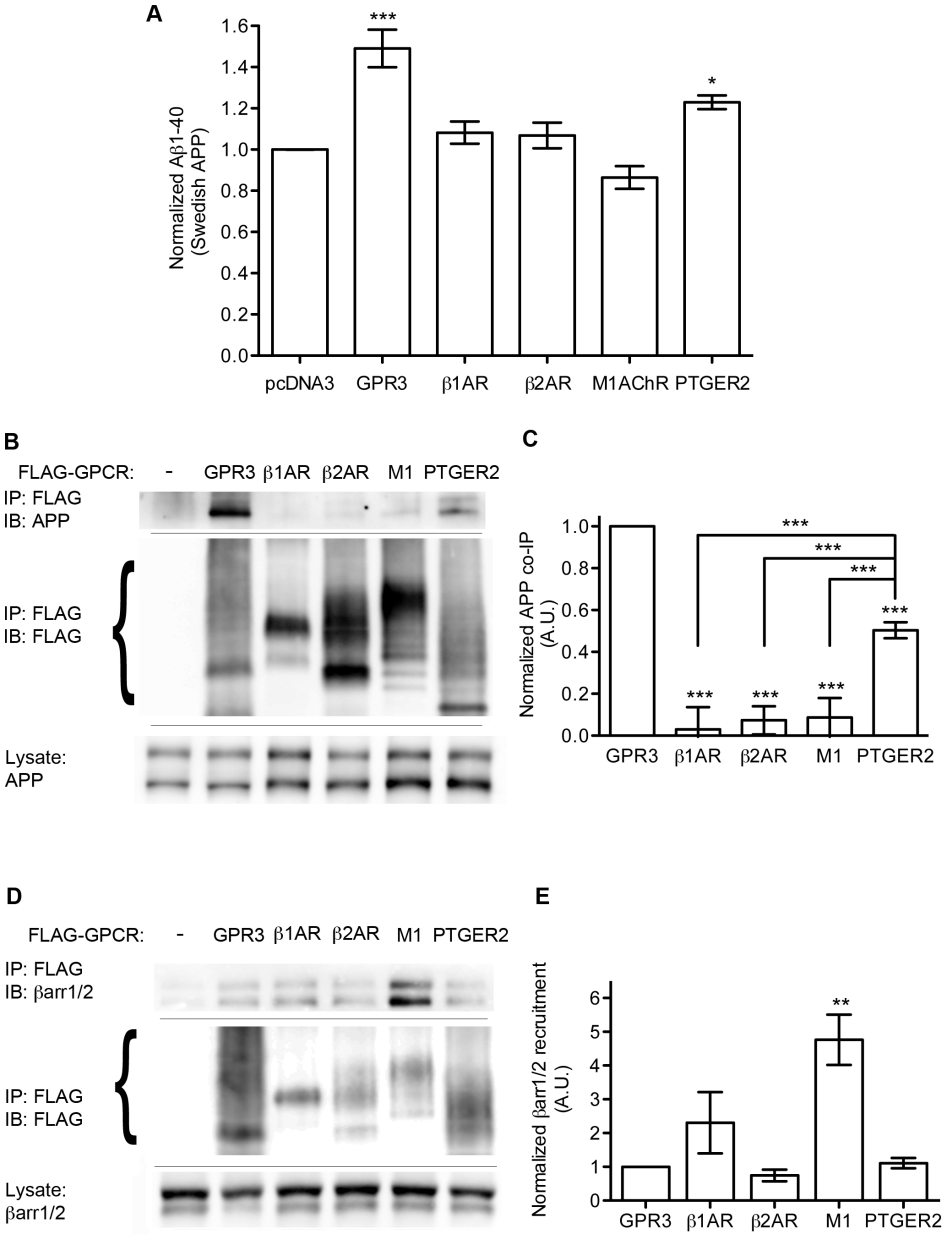
GPCR-APP complex formation correlates with receptor-stimulated Aβ production. A) Aβ1-40 ELISA data from SweAPP-HEK cells transfected with FLAG-tagged GPCRs as indicated. n = 8, 9, 7, 6, 7 and 8 from left to right. Statistical significance was determined by one-way ANOVA with a Dunnett test comparing all columns with vector control. (*p<0.05, ***p<0.001). B). Co-IP of APP with FLAG-GPCRs transfected as indicated in SweAPP-HEK cells. Braces indicate the observed MW range for the transfected GPCRs. C). Quantification (by densitometry) of APP co-IP with FLAG-tagged GPCRs. n = 4, 3, 4, 4 and 8 from left to right. Statistical analyses were performed by one-way ANOVA with a Bonferroni post-hoc test comparing all columns with GPR3 and selected comparisons as indicated (***p<0.001). D). Representative Western blots of endogenous β-arrestin co-IP with FLAG-GPCRs from unstimulated SweAPP-HEK cells. E). Quantification (by densitometry) of β-arrestin co-IP with FLAG-GPCRs. n = 3 for all conditions. Statistical significance was determined by one-way ANOVA with a Dunnett test comparing all columns with wild-type GPR3. (**p<0.01).

The discovery that APP may selectively interact with some GPCRs, like GPR3, but not others, such as the β2AR, led us to screen a broader panel of GPCRs for Aβ production and APP interactions (Figure 5). We transfected FLAG-tagged GPR3, β1-adrenergic receptor (β1AR), β2AR, M1 muscarinic acetylcholine receptor (M1AChR), prostaglandin receptor PTGER2, or empty vector into SweAPP-HEK cells, first looking at the ability of these receptors to stimulate Aβ production under basal culture conditions (Figure 5A). Testing the culture supernatant 3 days post-transfection, only GPR3 (1.49+/−0.09 fold relative to control) and PTGER2 (1.23+/−0.03 fold) showed significant increases in Aβ, while the β1AR (1.08+/−0.05), β2AR (1.07+/−0.06), and M1AChR (0.86+/−0.06) were not significantly different from vector control. We find this dichotomy extends to the GPCR-APP complex as well (Figure 5B–C). APP co-immunoprecipitated with GPR3, but not with β1AR, β2AR and M1AChR, which did not enhance Aβ production. The degree of co-IP of APP with PTGER2 was 46+/−3% of that seen with GPR3, which corresponds with the more modest enhancement of Aβ production by PTGER2. However, when we examined the receptor IPs for β-arrestin recruitment, we found that M1AChR (4.77+/−.75 fold relative to GPR3) and β1AR (2.31+/0.91) showed the highest levels of β-arrestin co-IP under non-stimulated cell culture conditions. Co-IP of endogenous β-arrestins for β2AR (0.75+/−0.17) and PTGER2 (1.11+/−0.15) were not significantly different from GPR3. Thus there is no correlation between interaction with endogenous β-arrestins and the ability of a GPCR to stimulate Aβ production. Rather, our data indicate that Aβ production is correlated with a GPCR’s interaction with APP.

**Figure 6:**
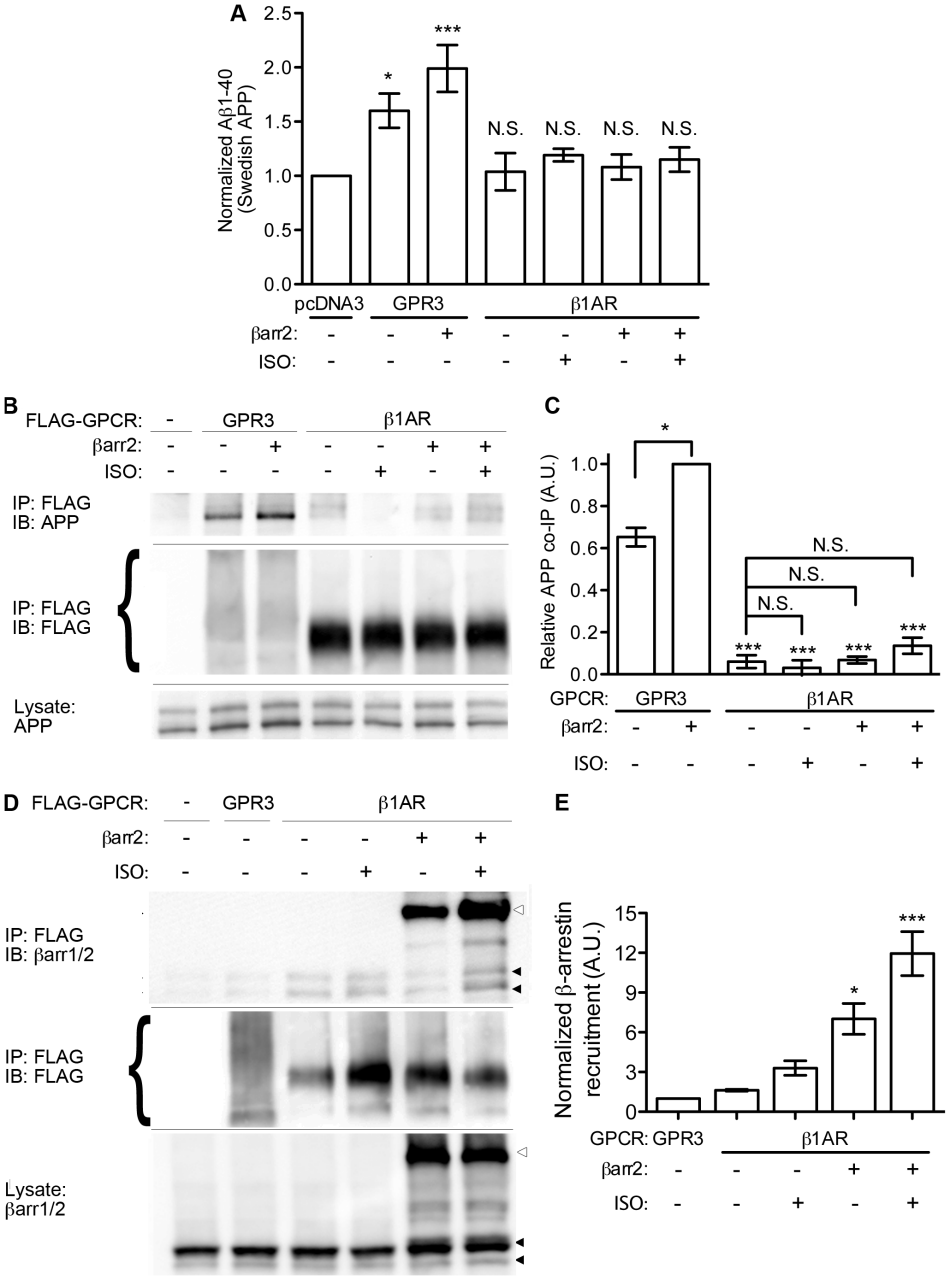
Agonist stimulation of β1AR promotes β-arrestin recruitment but not interaction with APP or Aβ production. A). Aβ1-40 ELISA from SweAPP-HEK cells transfected with FLAG-GPR3 or FLAG-β1AR and βarr2-EGFP and treated with isoproterenol (ISO, 10 μM, 30 min) as indicated. n = 3 for all conditions. Statistical significance was determined by one-way ANOVA with a Dunnett post-hoc test comparing all treatments with unstimulated vector control. (*p<0.05, ***p<0.001). B). APP co-IP with FLAG-GPR3 or FLAG-β1AR from SweAPP-HEK cells transfected and treated as indicated. C). Quantification (by densitometry) of APP co-IP with FLAG-GPCR IPs. n = 3, 3, 3, 3, 6 and 6 from left to right. Statistical significance was determined by one-way ANOVA with a Bonferroni post-hoc test comparing all conditions with GPR3+βarr2 and select comparisons as indicated. (*p<0.05, ***p<0.001). D). β-arrestin co-IP with FLAG-GPR3 or FLAG-β1AR from SweAPP-HEK cells transfected and treated as indicated. Open arrowheads indicate transfected βarr2-EGFP. Closed arrowheads mark endogenous β-arrestin1/2. E). Quantification (by densitometry) of co-immunoprecipitated β-arrestins with FLAG-GPR3 or FLAG-β1AR. n = 3 for all treatments. Statistical significance was determined by one-way ANOVA with a Dunnett post-hoc test comparing all treatments with unstimulated vector control. (*p<0.05, ***p<0.001).

We also considered the possibility that agonist stimulation may be necessary for some GPCRs to promote an interaction with APP and promote its processing. To this end, we utilized the β1AR and looked at Aβ levels, β-arrestin recruitment and APP co-IP in the presence or absence of 10 μM isoproterenol stimulation for 30 minutes (Figure 6). Consistent with the experiments above, GPR3 potentiated Aβ production (1.49+/−0.27 fold relative to vector control) in the SweAPP cell line, but β1AR had no significant effect (1.08+/−0.14); this lack of effect did not change with isoproterenol stimulation of the receptor (1.19+/−0.10) (Figure 6A). Moreover, overexpression of βarr2 did not enhance Aβ production when co-transfected with β1AR in either the unstimulated (1.08+/−0.20) or isoproterenol-stimulated conditions (1.15+/−0.20). When we examined the receptor IPs for interaction with APP, only GPR3 (0.65+/−0.08 fold relative to GPR3+βarr2) and the GPR3+βarr2 co-transfected samples showed substantial co-IP with APP (Figure 6B, C). We observed a β1AR-β-arrestin interaction under basal conditions (1.63+/−0.07 fold over GPR3) that increased with agonist stimulation of the β1AR (3.30+/−0.54) (Figure 6D). As expected, overexpression of βarr2-EGFP robustly increased the amount of total β-arrestin in the β1AR receptor co-IP (7.02+/−1.16) and the difference increased even further when β1AR was co-transfected with βarr2 and stimulated with isoproterenol (11.94+/−1.65) (Figure 6D, E). Thus for the β1AR, a prototypic GPCR, agonist stimulation and βarr2 binding do not correlate with interaction with APP or elevation of Aβ levels.

**Figure 7:**
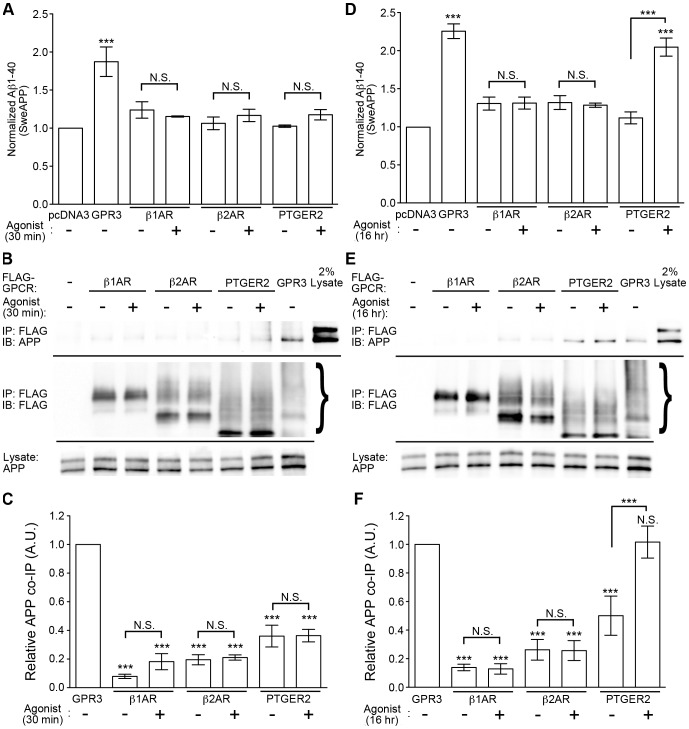
PGE2 promotes Aβ production and PTGER2-APP interaction at 16 hours stimulation. A). Aβ1-40 ELISA data from SweAPP-HEK cells transfected with βarr2-EGFP and empty vector or FLAG-tagged GPCRs as indicated. After 48 hours, the cells were serum starved in fresh media and stimulated for 30 minutes with either 10 μM isoproterenol (β1AR, β2AR) or 10 μM PGE2 (PTGER2). Data for β1AR+βarr2−/+ ISO are re-plotted from Fig. 6A. n = 4, 4, 4, 4, 4, 4, 3, and 3 from left to right. Statistical significance was determined by one-way ANOVA with a Bonferroni post-hoc test comparing all treatments with unstimulated vector control and select comparisons as indicated. (***p<0.001). B). APP co-IP with FLAG-GPCRs from SweAPP-HEK cells transfected and stimulated for 30 min as indicated. C). Quantification (by densitometry) of APP co-immunoprecipitated with FLAG-GPCR IPs. Data for β1AR+βarr2−/+ ISO are re-plotted from Fig. 6C. n = 5, 5, 5, 6, 6, 6 and 6 from left to right. Statistical significance was determined by one-way ANOVA with a Bonferroni post-hoc test comparing all conditions with GPR3+βarr2 and select comparisons as indicated. (***p<0.001). D). Aβ1-40 ELISA from SweAPP-HEK cells transfected with βarr2-EGFP and empty vector or FLAG-tagged GPCRs as indicated. After 48 hours, the cells were serum starved in fresh media and stimulated for 16 hours with either 10 μM isoproterenol (β1AR, β2AR) or 10 μM PGE2 (PTGER2). n = 5, 5, 3, 3, 4, 4, 5, and 5 from left to right. Statistical significance was determined by one-way ANOVA with a Bonferroni post-hoc test comparing all treatments with unstimulated vector control and select comparisons as indicated. (***p<0.001). E). APP co-IP with FLAG-GPCRs from SweAPP-HEK cells transfected and stimulated for 16 hours as indicated. F). Quantification (by densitometry) of APP co-immunoprecipitated with FLAG-GPCR IPs. n = 4 for all conditions. Statistical significance was determined by one-way ANOVA with a Bonferroni post-hoc test comparing all conditions with GPR3+βarr2 and select comparisons as indicated. (***p<0.001).

What is the effect of agonist stimulation of a receptor like PTGER2, which does stimulate APP processing under basal conditions (Figure 5A)? To address this question, we transfected SweAPP cells with βarr2-EGFP and co-transfected with empty vector, GPR3, β1AR, β2AR or PTGER2. The cultures were serum starved and then stimulated for 30 minutes with their respective agonists (10 μM isoproterenol for beta-adrenergic receptors, or 10 μM PGE2 for PTGER2). However, under these conditions, only GPR3 (1.87+/−0.39 fold over vector control) promoted a significant increase in Aβ levels (Figure 7A). At 30 min of agonist stimulation, we also saw minimal interaction of β1AR, β2AR or PTGER2 with APP and no agonist effects in receptor co-IP experiments (Figure 7B, C). However, when transfected cells were stimulated for a prolonged period with agonist (16 hours), we found that PTGER2-transfected cells stimulated with PGE2 (2.06+/−0.27) increased Aβ to levels comparable to GPR3 (2.27+/−0.22) (Figure 7D). Prolonged PGE2 stimulation also potentiated the biochemical association of PTGER2 with APP (Figure 7E, F). No such effect was observed for either β1AR or β2AR with isoproterenol. Collectively, these data indicate that agonist stimulation can promote APP interaction and APP processing for a subset of GPCRs like PTGER2. On the other hand, GPR3, which has high constitutive activity, can associate with APP and enhance Aβ production in the absence of an exogenous agonist.

**Figure 8:**
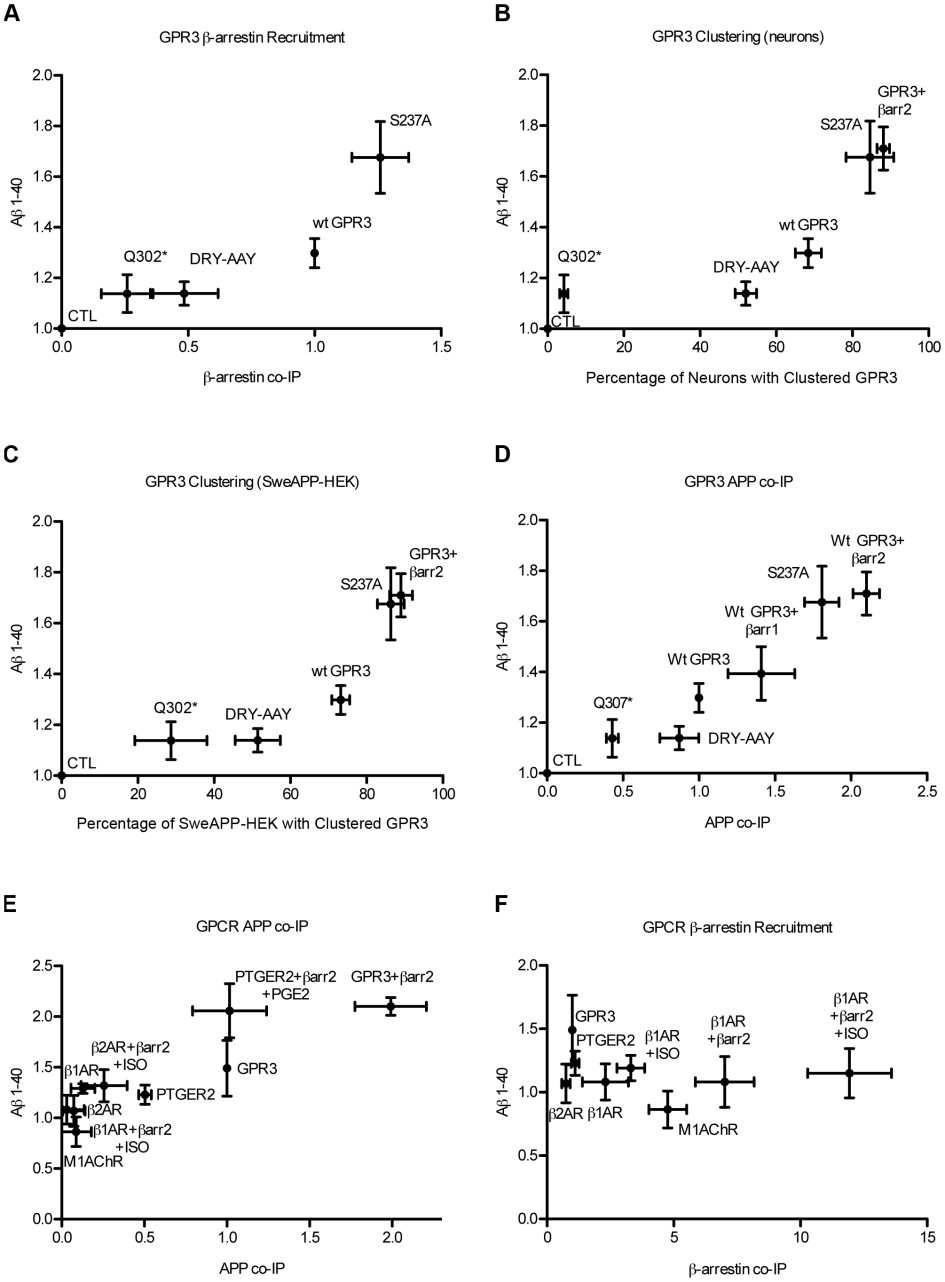
Correlation graphs of factors involved in GPCR-stimulated APP processing. A). Aβ levels (normalized to vector control) plotted as a function of GPR3 recruitment of endogenous β-arrestins in SweAPP HEK cells. B). Aβ production (from SweAPP-HEK) as a function of GPR3 clustering in neurons. C). Aβ production as a function of GPR3 clustering in SweAPP-HEK cells. D). Aβ production of wild-type GPR3 and GPR3 mutants as a function of APP co-IP. E). Aβ1-40 produced by the GPCR panel as a function of receptor interaction with APP. F). Aβ levels of the GPCR panel plotted as a function of β-arrestin recruitment.

Looking across all parameters measured, we examined the correlations of our data sets to better understand the mechanisms of GPCR-stimulated APP processing (Figure 8). For wild-type GPR3 and the GPR3 mutants, Aβ production was positively correlated with β-arrestin recruitment to the receptors (Figure 8A), with intracellular clustering of the receptor in neurons and in SweAPP-HEK cells (Figure 8B,C), and with co-IP of APP with the receptor (Figure 8D). However, when we look at the broader panel of GPCRs, APP co-immunoprecipitation with the GPCR correlates positively with stimulation of Aβ production, whereas β-arrestin recruitment is non-correlative (Figure 7E,F). Further, as expected for GPCR internalization, the GPR3 clustering in endosomes is also correlated with the ability of the receptor to bind β-arrestins (Figure S4). Thus, we conclude that the ability of GPCRs to stimulate Aβ production is related to their capability to interact with APP and that β-arrestin recruitment, while necessary, is not sufficient to drive GPCR-stimulated processing of APP.

## Discussion

Here we report that a GPCR-APP complex is formed by GPR3 and agonist-stimulated PTGER2 (GPCRs that potentiate APP processing), but not formed by receptors that do not affect Aβ production (such as β1AR or M1AChR). This selective interaction offers a resolution to the question: How can βarr2 be integral to GPCR-mediated Aβ production, when it interacts nearly universally with GPCRs, and yet only a subset of GPCRs enhances APP cleavage? Our data support the idea that βarr2 is crucial for GPCR-mediated enhancement of APP processing, but they suggest that formation of a GPCR-APP complex is also fundamental for receptor-stimulated Aβ generation. The degree of the GPCR-APP interaction more closely reflects a receptor’s Aβ production potential than does β-arrestin binding, and thus formation of a receptor-APP complex defines a new subclass of GPCRs that can promote Aβ processing.

### Relation to other Studies of GPCR-stimulated Aβ Production

Several reports have described stimulation of Aβ production by GPCRs including PTGER2 [37], the serotonin receptor 5HTR2C [38], thyrotropin-releasing hormone receptor [39], and the α_2a_-adrenergic receptor [39]. Thathiah et al discovered GPR3-stimulated APP processing in a screen for modulators of Aβ production, showing GPR3 stimulates γ-secretase activity via signaling pathways independent of the receptor’s constitutive G_s_ activity [14]. More recently, the same group found elevated βarr2 levels in human AD tissue samples, and moreover, βarr2 knockout mice, but not βarr1 knockouts, showed lower Aβ levels in the hippocampus and cortex when crossed with an AD mouse model [36]. These findings agree with our data that βarr2 overexpression potentiates, and βarr2 knockdown mitigates, the production of Aβ in SweAPP cells (Figure 1A–B). However, βarr2 shRNA knockdown did not significantly reduce Aβ production in WtAPP cells (Figure 1F). One explanation is that βarr2 protein has a relatively long half-life of 12–15 h [40], thus the knockdown of endogenous βarr2 protein is likely to be incomplete during the time period when GPR3 is co-transfected and expressed. In a genetic experiment, Thathiah et al [36] showed that GPR3 does not increase Aβ production in βarr2 knockout neurons, which strongly supports the idea that βarr2 is necessary for GPR3-stimulated Aβ production.

One proposed molecular mechanism for GPR3 stimulation of APP processing is through β-arrestin2-mediated recruitment of active γ-secretase complex via an interaction with the Aph-1a subunit [36]. This model is consistent with reports of internalization-dependent, G protein-independent increases in γ-secretase activity induced by the β2AR [41] and δ-opioid receptor [42]. Though β-arrestins are not specifically investigated in these studies, a role for βarr2-mediated internalization and recruitment of γ-secretase to the activated GPCRs fits the data. A notable discrepancy is that in our cells, possibly due to differences in the experimental systems, β2AR does not enhance Aβ production. We see a synergistic effect of GPR3 and βarr2 on Aβ production for SweAPP, but not WtAPP (Figure 1B,E). This finding supports the hypothesis that the effect of βarr2 is to enhance γ-secretase activity, because for SweAPP (a much better substrate for beta-secretase) γ-secretase is more rate limiting – in contrast to wild-type APP, for which BACE cleavage is rate limiting.

### Molecular Mechanisms of the GPR3 Mutants

Data from our GPR3 mutants implicate the formation of a receptor-APP complex and internalization by βarr2 as processes involved in GPR3-stimulated Aβ production. However, the mechanisms by which these mutations alter β-arrestin recruitment and APP binding are not completely understood. Only the Q302* cytoplasmic tail truncation mutant, intended to reduce interactions with β-arrestins, behaved as initially hypothesized. The S237A mutant was created to remove a putative GRK phosphorylation site and thereby reduce β-arrestin recruitment. Instead, this mutant showed enhanced β-arrestin binding, along with stronger stimulation of Aβ production and enhanced co-IP of APP. The fact that Aβ production by S237A could not be further enhanced by βarr2 overexpression is consistent with the idea that this mutant binds endogenous β-arrestins so well that this interaction is no longer limiting. S237A may promote a receptor conformation favoring β-arrestin binding, or alternatively, this residue may be a site of inhibitory phosphorylation that is relieved by the S237A mutation.

Multiple lines of evidence indicate GPR3-stimulated Aβ production is G protein-independent [14]. Thus, we expected that DRY-AAY, a mutation that reduces G protein coupling, would exhibit Aβ production equivalent to wild-type GPR3. Instead, the DRY-AAY mutant showed diminished stimulation of Aβ production, which correlated well with reduced β-arrestin recruitment and reduced intracellular clustering of this mutant. Further complicating the issue, Thathiah et al also used a DRY-AAY mutant and observed enhanced β-arrestin binding and Aβ production [36]. This discrepancy may be due to differences in the respective SweAPP-HEK cell lines. However, both reports agree that Aβ production by mutant GPR3 receptors correlates with β-arrestin binding.

### Implications of GPCR-APP Complex Formation

We find the formation of a protein complex including GPR3 and APP is associated with enhanced Aβ production and this is the only parameter tested that correlates with APP processing across multiple GPCRs. While this interaction may define a new subset of Aβ-modulating GPCRs, critical questions about the nature of this complex remain to be addressed. First, does APP interact directly or indirectly with GPCRs such as GPR3? Future experiments using mass spectrometry of APP immunoprecipitates may identify additional GPCRs or regulators of the receptor-APP protein complex. Unfortunately, because of a lack of good antibodies and low endogenous expression of GPR3, our study and many others rely on epitope-tagged GPCRs, whose overexpression may lead to artifactual interactions. Second, what are the specific domains and residues required for GPCRs to interact with APP? Experimentally, this could be addressed with chimeric receptors made from GPR3 and a receptor such as β1AR that does not stimulate Aβ production. Finally, what is the specific role for β-arrestin in the receptor-APP interaction? Does it participate as a scaffold in the formation of a receptor-βarr2-APP ternary complex, or does the β-arrestin-dependent trafficking of GPCRs like GPR3 into endosomes enrich the local concentrations of receptor and APP, promoting their interaction? Experiments in βarr2 knockout cells should verify whether βarr2 is required for GPR3-APP binding. Finally, we note that PGE2 failed to stimulate APP-PTGER2 association at 30 minutes, a time point when GPCR endocytosis should be occurring. This suggests the APP-receptor complex may be created in the endosomes after internalization, rather than forming at the plasma membrane. More detailed PGE2 time courses with and without inhibition of endocytosis may provide insights into the timing and processes required to form the PTGER2-APP complex. Ultimately, these experiments will provide a deeper understanding of the molecular nature of the GPCR-APP interaction and potentially offer new targets for therapeutic intervention in AD.

## Supporting Information

Figure S1 **The DRY-AAY mutation impairs GPR3-stimulated cAMP production.** SweAPP-HEK cells were transfected with either empty vector (pcDNA3), GPR3, or DRY-AAY. Two days later, the cells were treated with 10 μM IBMX for 30 minutes to inhibit phosphodiesterases. The cells were lysed and cAMP accumulation was quantified using an HTRF ELISA kit. n = 3, 4 and 3 from left to right. Statistical significance was determined by one-way ANOVA with a Bonferroni post-hoc test, comparing all columns. (*p<0.05, **p<0.01).
(TIF)

Figure S2 **GPR3 clusters partially co-localize with endogenous β-arrestins and the endosomal marker EEA1.** Dissociated rat hippocampal neurons were grown on glass coverslips and prepared as described in Materials and Methods. The cells were incubated with anti-FLAG and either A) anti-β-arrestin1/2 or B) anti-EEA1 (middle panel) primary antibodies as indicated. Representative confocal images are shown. Merged images show FLAG in the red channel and either β-arrestin1/2 or EEA1 in green, with yellow pixels indicating colocalization.
(TIF)

Figure S3 **APP co-immunoprecipitates with GPR3, but not with β-arrestin1/2.** SweAPP-HEK cells were transfected with empty vector, βarr1-EGFP, βarr2-EGFP or FLAG- GPR3 as shown and FLAG- or EGFP-immunoprecipitations were blotted for co-immunoprecipitated APP (upper) and levels of immunoprecipitation for the bait proteins (lower).
(TIF)

Figure S4 **GPR3 clustering is a function of β-arrestin recruitment.** Correlation graph showing the fraction of SweAPP-HEK cells transfected with the indicated GPR3 mutants in a clustered staining pattern as a function of co-IP with endogenous β-arrestins.
(TIF)
