# Supplementary figures and images for: GPR3 Stimulates Aβ Production via Interactions with APP and β-Arrestin2
Source: PLoS One. 2013 Sep 12;8(9):e74680. doi: 10.1371/journal.pone.0074680 (PMC3771882; doi:10.1371/journal.pone.0074680)

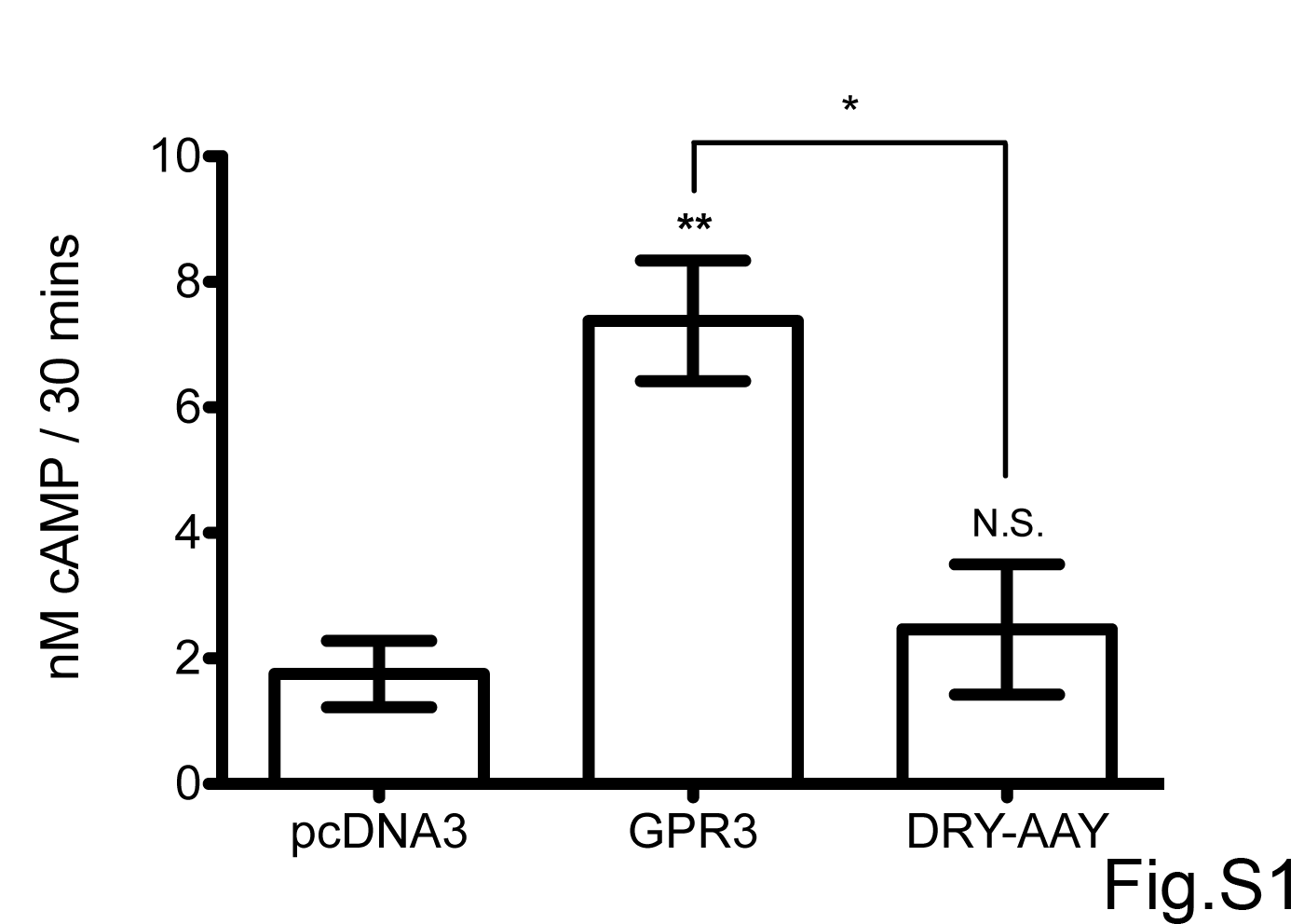

Supplement: Figure S1 — The DRY-AAY mutation impairs GPR3-stimulated cAMP production. SweAPP-HEK cells were transfected with either empty vector (pcDNA3), GPR3, or DRY-AAY. Two days later, the cells were treated with 10 μM IBMX for 30 minutes to inhibit phosphodiesterases. The cells were lysed and cAMP accumulation was quantified using an HTRF ELISA kit. n = 3, 4 and 3 from left to right. Statistical significance was determined by one-way ANOVA with a Bonferroni post-hoc test, comparing all columns. (*p<0.05, **p<0.01). (TIF) [file pone.0074680.s001.tif]

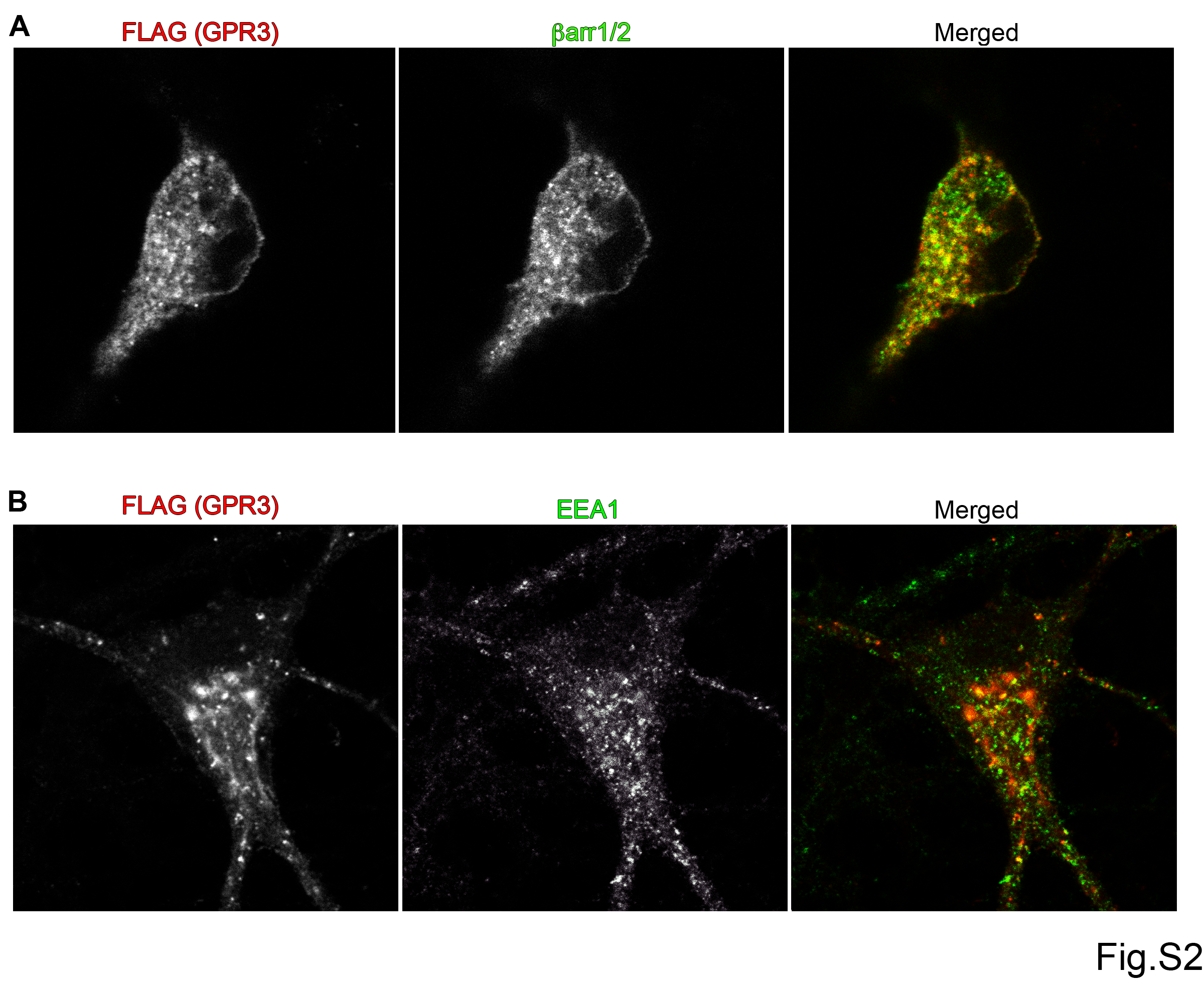

Supplement: Figure S2 — GPR3 clusters partially co-localize with endogenous β-arrestins and the endosomal marker EEA1. Dissociated rat hippocampal neurons were grown on glass coverslips and prepared as described in Materials and Methods. The cells were incubated with anti-FLAG and either A) anti-β-arrestin1/2 or B) anti-EEA1 (middle panel) primary antibodies as indicated. Representative confocal images are shown. Merged images show FLAG in the red channel and either β-arrestin1/2 or EEA1 in green, with yellow pixels indicating colocalization. (TIF) [file pone.0074680.s002.tif]

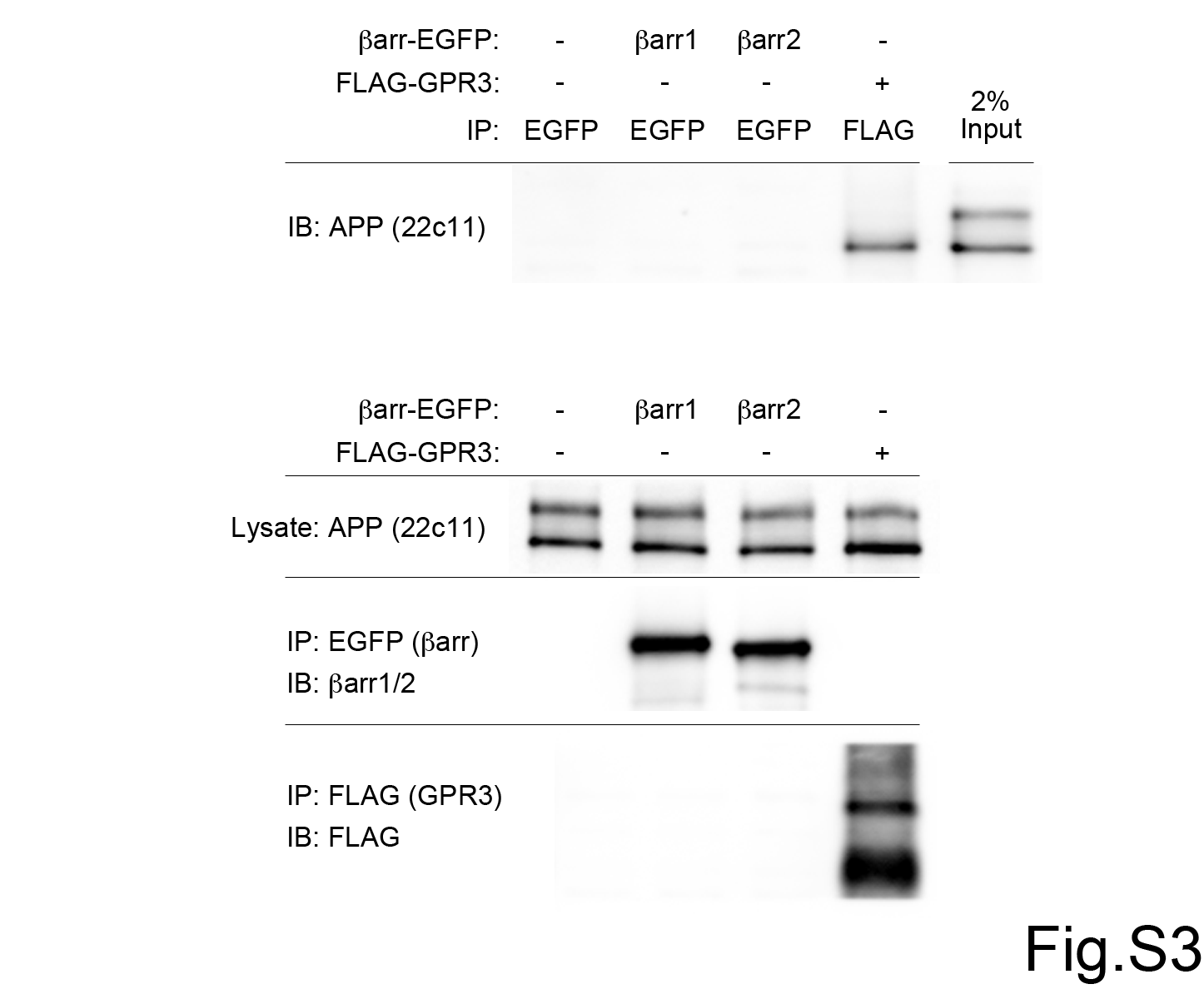

Supplement: Figure S3 — APP co-immunoprecipitates with GPR3, but not with β-arrestin1/2. SweAPP-HEK cells were transfected with empty vector, βarr1-EGFP, βarr2-EGFP or FLAG- GPR3 as shown and FLAG- or EGFP-immunoprecipitations were blotted for co-immunoprecipitated APP (upper) and levels of immunoprecipitation for the bait proteins (lower). (TIF) [file pone.0074680.s003.tif]

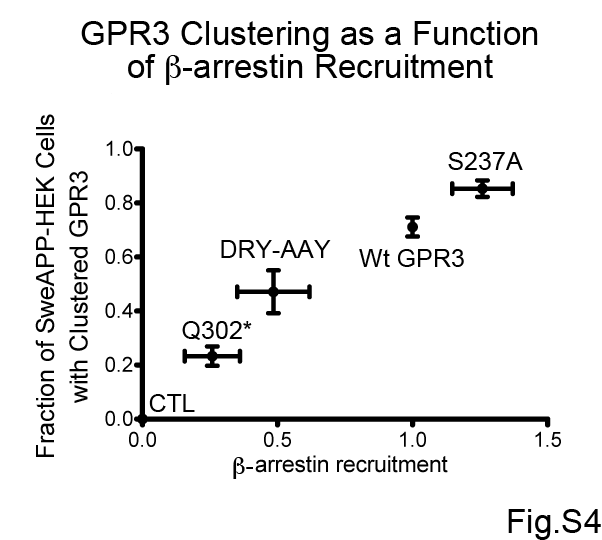

Supplement: Figure S4 — GPR3 clustering is a function of β-arrestin recruitment. Correlation graph showing the fraction of SweAPP-HEK cells transfected with the indicated GPR3 mutants in a clustered staining pattern as a function of co-IP with endogenous β-arrestins. (TIF) [file pone.0074680.s004.tif]
